# Intracellular Nitrate of Marine Diatoms as a Driver of Anaerobic Nitrogen Cycling in Sinking Aggregates

**DOI:** 10.3389/fmicb.2016.01669

**Published:** 2016-11-01

**Authors:** Anja Kamp, Peter Stief, Laura A. Bristow, Bo Thamdrup, Ronnie N. Glud

**Affiliations:** ^1^AIAS, Aarhus Institute of Advanced Studies, Aarhus UniversityAarhus, Denmark; ^2^Department of Biology and Nordic Center for Earth Evolution, University of Southern DenmarkOdense, Denmark; ^3^Department of Biogeochemistry, Max Planck Institute for Marine MicrobiologyBremen, Germany; ^4^Department of Biogeochemistry and Earth Science, Scottish Association for Marine ScienceOban, UK; ^5^Department of Bioscience, Arctic Research Centre, Aarhus UniversityAarhus, Denmark

**Keywords:** nitrate respiration, denitrification, DNRA, nitrogen loss, low-oxygen environments, marine snow, stable isotopes

## Abstract

Diatom-bacteria aggregates are key for the vertical transport of organic carbon in the ocean. Sinking aggregates also represent pelagic microniches with intensified microbial activity, oxygen depletion in the center, and anaerobic nitrogen cycling. Since some of the aggregate-forming diatom species store nitrate intracellularly, we explored the fate of intracellular nitrate and its availability for microbial metabolism within anoxic diatom-bacteria aggregates. The ubiquitous nitrate-storing diatom *Skeletonema marinoi* was studied as both axenic cultures and laboratory-produced diatom-bacteria aggregates. Stable ^15^N isotope incubations under dark and anoxic conditions revealed that axenic *S. marinoi* is able to reduce intracellular nitrate to ammonium that is immediately excreted by the cells. When exposed to a light:dark cycle and oxic conditions, *S. marinoi* stored nitrate intracellularly in concentrations >60 mmol L^-1^ both as free-living cells and associated to aggregates. Intracellular nitrate concentrations exceeded extracellular concentrations by three orders of magnitude. Intracellular nitrate was used up within 2–3 days after shifting diatom-bacteria aggregates to dark and anoxic conditions. Thirty-one percent of the diatom-derived nitrate was converted to nitrogen gas, indicating that a substantial fraction of the intracellular nitrate pool of *S. marinoi* becomes available to the aggregate-associated bacterial community. Only 5% of the intracellular nitrate was reduced to ammonium, while 59% was recovered as nitrite. Hence, aggregate-associated diatoms accumulate nitrate from the surrounding water and sustain complex nitrogen transformations, including loss of fixed nitrogen, in anoxic, pelagic microniches. Additionally, it may be expected that intracellular nitrate not converted before the aggregates have settled onto the seafloor could fuel benthic nitrogen transformations.

## Introduction

The oceans teem with diatoms that can form huge phytoplankton blooms in surface layers (Leblanc et al., 2012; Simon et al., 2014). In the wake of these blooms, diatom-bacteria aggregates can form in masses and sink out of the photic zone toward the sea floor sustaining the benthic communities (Smetacek, 1985; Thornton, 2002). In polar regions, aggregates can also form at high abundance as sea ice melting leads to mass release of algae from brine channels and the underside of ice floes (Boetius et al., 2013; Fernández-Méndez et al., 2014; Glud et al., 2014; Boetius et al., 2015). Natural diatom-derived aggregates consist of a diverse assemblage of diatoms, the bacterial and archaeal community of surface waters (Thiele et al., 2015), viruses, other planktonic organisms, and detritus (Simon et al., 2002). Sinking organic aggregates, also named “marine snow,” are extensively studied, often with a focus on the vertical transport of organic carbon to the seafloor by the “biological pump” (e.g., Riley, 1963; Silver et al., 1978; Shanks and Trent, 1979; Grossart et al., 1998; Simon et al., 2002; Turner, 2015). In contrast, organic nitrogen compounds are preferentially degraded during the sinking process, which increases the C/N ratio of aggregates during their descent (Martin et al., 1987; Smith et al., 1992; Dang and Lovell, 2016). The vertical transport of intracellularly stored ${NO}_{3}^{-}$ within sinking diatom-bacteria aggregates (Stief et al., 2016) has so far not been documented *in situ*. To date, diatoms are the only known ${NO}_{3}^{-}$-storing microorganisms in marine snow, e.g., the pelagic species *Skeletonema marinoi* and *Thalassiosira weissflogii* (Kamp et al., 2011, 2013; Stief et al., 2016). Both genera are very abundant in the ocean and can contribute significantly to spring blooms and subsequent aggregate formation (Bresnan et al., 2009; Degerlund and Eilertsen, 2010; Leblanc et al., 2012).

The pelagic, centric *T. weissflogii* as well as the benthic, pennate *Amphora coffeaeformis* are known to use intracellular ${NO}_{3}^{-}$ for dissimilatory nitrate reduction to ammonium (DNRA; ${NO}_{3}^{-}$ → ${NO}_{2}^{-}$ → ${NH}_{4}^{+}$) after sudden shifts to dark and anoxic conditions (Kamp et al., 2011, 2013, 2015), which might also hold true for *S. marinoi*. DNRA is an anaerobic nitrate reduction pathway, which can be used to conserve energy in the absence of O_2_ (Kraft et al., 2011; Thamdrup, 2012). Instead of being used as terminal electron acceptor in anaerobic respiration, intracellular ${NO}_{3}^{-}$ may also serve as an electron sink for fermentation processes in diatoms exposed to anoxic conditions. This process would also produce ${NH}_{4}^{+}$ that is eventually excreted by the cell, similar to “ammonia fermentation” in fungi (Zhou et al., 2002; Stief et al., 2014). Other energy-providing pathways of the anaerobic nitrogen cycle being of potential importance in diatom-bacteria aggregates include dissimilatory nitrate reduction to nitrite (DNRN; ${NO}_{3}^{-}$ → ${NO}_{2}^{-}$), denitrification (${NO}_{3}^{-}$ → ${NO}_{2}^{-}$ → NO→ N_2_O → N_2_), incomplete denitrification (${NO}_{3}^{-}$ → ${NO}_{2}^{-}$ → NO→ N_2_O), and anammox (${NO}_{2}^{-}$ + ${NH}_{4}^{+}$ → N_2_) (Stief et al., 2016).

Here, we focus on metabolic pathways of dissimilatory nitrate reduction performed by microorganisms exposed to anoxic environmental conditions. Diatom-bacteria aggregates, and “marine snow” in general, can be exposed to such conditions while sinking through oxygen-depleted water masses of oxygen minimum zones (OMZ’s; Ploug and Bergkvist, 2015; Stief et al., 2016). However, even in oxic settings, enhanced microbial activity may facilitate the formation of central anoxia in sinking aggregates (Ploug et al., 1997; Klawonn et al., 2015; Stief et al., 2016). Indeed, DNRA has previously been observed in large aggregates exposed to ${NO}_{3}^{-}$ concentrations of 25–30 μmol L^-1^ in the surrounding seawater and to ambient O_2_ levels corresponding to 30–40% air saturation (Klawonn et al., 2015; Stief et al., 2016). Intracellular ${NO}_{3}^{-}$ stored by diatoms may, however, serve as a ${NO}_{3}^{-}$ source for dissimilatory nitrate reduction within diatom-bacteria aggregates, providing independence from external ${NO}_{3}^{-}$ supply and also allowing for respiratory organic carbon mineralization in the absence of O_2_ (Stief et al., 2016).

This study aims to reveal (a) whether the ubiquitous, aggregate-forming diatom *S. marinoi* is able to perform DNRA under dark and anoxic conditions, (b) whether and how fast the ${NO}_{3}^{-}$ stored intracellularly by aggregate-associated *S. marinoi* is used after shifting diatom-bacteria aggregates to dark and anoxic conditions, and (c) to what extent anoxic diatom-bacteria aggregates release ${NH}_{4}^{+}$ (from diatom-DNRA) into the surrounding water relative to other products of anaerobic nitrogen cycling inside the aggregates.

## Materials and Methods

### Strain and Cultivation

An axenic strain of the marine pelagic, chain-forming, diatom *S. marinoi* (CCMP 1332) was obtained from the Provasoli-Guillard National Center for Marine Algae and Microbiota (NCMA; formerly CCMP). This strain was formerly referred to as *S. costatum* and has previously been studied for its intracellular ${NO}_{3}^{-}$ storage capacity (Kamp et al., 2011). The diatoms were cultured in F/2 medium plus silicate (Guillard and Ryther, 1962) prepared with filtered (0.45 μm) and autoclaved Baltic Sea water (salinity adjusted to 30). The cultivation temperature was 14°C, the light:dark cycle was 10:14 h, and the light intensity was 20 μmol photons m^-2^ s^-1^. *S. marinoi* was frequently checked for possible contaminations with bacteria by careful phase-contrast microscopy and by plating out subsamples of the cultures on nutrient agar plates. Contamination of the cultures was not detected at any point during the present study.

### Dissimilatory Nitrate Reduction by Axenic *Skeletonema marinoi*

*Skeletonema marinoi* was investigated for possible pathways of dissimilatory ${NO}_{3}^{-}$ reduction by ^15^N- stable isotope labeling of its intracellular ${NO}_{3}^{-}$ pool and following the time course of the intracellular ^15^${NO}_{3}^{-}$ concentration and the extracellular ^15^${NH}_{4}^{+}$, ^15^${NO}_{2}^{-}$, N_2_O, and ^15^N_2_ concentrations in axenic *S. marinoi* cultures.

To prepare the *S. marinoi* cells for the experiment, their non-labeled intracellular ${NO}_{3}^{-}$ (i.e., intracellular ^14^${NO}_{3}^{-}$) was replaced with ^15^N-labeled intracellular ${NO}_{3}^{-}$ (i.e., intracellular ^15^${NO}_{3}^{-}$). The cells were washed three times with sterile NaCl solution (salinity 30; 5 min; 600 *g*) to remove ^14^${NO}_{3}^{-}$ from the growth medium, flushed with N_2_ for 30 min to remove O_2_ (the O_2_ concentration was followed with optode spots; SensorSpot, Pyroscience, Germany), and incubated for 24 h in ${NO}_{3}^{-}$-free F/2 medium plus silicate, to make the cells use up their intracellular ^14^${NO}_{3}^{-}$. After this “starvation procedure,” the cells were washed again and grown for 12 h in F/2 medium plus silicate, in which ^14^${NO}_{3}^{-}$ was replaced with ^15^${NO}_{3}^{-}$ (50 μmol L^-1^; 98 atom %, Cambridge Isotope Laboratories), for subsequent accumulation of intracellular ^15^${NO}_{3}^{-}$.

For the experimental incubation, the concentrations of ${NH}_{4}^{+}$ (added to meet assimilation requirements) and sodium acetate (added as an electron donor) were adjusted to 100 μmol L^-1^ each in the growth medium and the *S. marinoi* culture was split in two.

(a) One half of the culture was transferred into a dark, gas-tight glass bottle, flushed with He for 30 min to introduce dark and anoxic conditions, thoroughly mixed, and distributed into 24 (+3) replicate 6-mL gas-tight incubation vials (Labco, UK) wrapped in aluminum foil. At time intervals of 0.5, 1, 1.5, 2, 3, 4, 5, and 6 h, a He headspace of 3 mL was set in each of three incubation vials, and the diatoms in the remaining 3 mL were killed with 100 μL ZnCl_2_ (50% w/v). The vials were stored upside-down at room temperature until measurement of ^15^N_2_ and N_2_O on a gas chromatography-isotopic ratio mass spectrometer (GC-IRMS; 184 Thermo Delta V Plus, Thermo Scientific; for details see Dalsgaard et al., 2012; Stief et al., 2016) and a gas chromatograph (GC 7890, Agilent Technologies), respectively. The cell suspensions collected during setting the headspace were filled into centrifugation tubes and centrifuged (5 min; 600 *g*). To calculate the intracellular ${NO}_{3}^{-}$ concentration, 25 μL of the well-mixed pellet was diluted 1:10 in NaCl solution (salinity 30) plus 4% formaldehyde for diatom cell counting in a Fuchs-Rosenthal counting chamber. The remaining pellet and 100 μL of the cell-free supernatant were separately frozen at -20°C for (intracellular) ${NO}_{3}^{-}$ analyses with an NO_x_ analyser (CLD 66s, EcoPhysics; for details see Kamp et al., 2011, 2013). The remaining cell-free supernatant was frozen at -20°C for measurement of ${NH}_{4}^{+}$, ^15^${NH}_{4}^{+}$, and ^15^${NO}_{2}^{-}$ concentrations (for details see Kamp et al., 2013; Stief et al., 2016). *S. marinoi* cells taken from three additional incubation vials were carefully investigated for bacterial contamination (see above) at the end of the experiment. No contamination was detected.

(b) The second half of the culture was kept under light and oxic conditions and sub-sampled at time intervals of 0, 1, 2, 3, 4, 5, and 6 h in triplicates each for the measurement of intracellular ${NO}_{3}^{-}$ and extracellular ^15^${NH}_{4}^{+}$ and total ${NH}_{4}^{+}$ concentrations.

Since the NO_x_ analyzer does not discriminate between ^14^${NO}_{3}^{-}$ and ^15^${NO}_{3}^{-}$, and the intracellular ^14^${NO}_{3}^{-}$ pool of *S. marinoi* might not have been completely depleted prior to the incubation with ^15^${NO}_{3}^{-}$ (see “starvation procedure”), intracellular ^15^${NO}_{3}^{-}$ was also measured with the cadmium/sulfamic acid assay in three samples taken at t_0_ (McIlvin and Altabet, 2005; Füssel et al., 2012). Together with the data obtained from the NO_x_ analyzer (i.e., ^14+15^${NO}_{3}^{-}$), the ^15^${NO}_{3}^{-}$ data were used to calculate the labeling fraction of the intracellular ${NO}_{3}^{-}$ pool at t_0_, which amounted to 52.5 ± 0.7%. This initial labeling fraction was assumed to remain constant throughout the experimental incubation because *S. marinoi* does not replenish the intracellular ${NO}_{3}^{-}$ pool under anoxia (**Supplementary Figure S1**) and isotope fractionation during passive ${NO}_{3}^{-}$ leakage from diatom cells is very unlikely. The initial labeling fraction of the intracellular ${NO}_{3}^{-}$ pool was therefore used to convert the measured ^15^N-concentrations to intracellular ${NO}_{3}^{-}$-derived N-concentrations (denoted as ^IC^N) by dividing the ^15^N-concentrations by 0.525.

### Intracellular Nitrate Storage by Free-Living and Aggregate-Associated *S. marinoi*

Free-living (axenic) *S. marinoi* cells as well as *S. marinoi* cells in diatom-bacteria aggregates were investigated for their ${NO}_{3}^{-}$ storage capacity under light and oxic conditions and its correlation to the extracellular ${NO}_{3}^{-}$ concentration, i.e., the ${NO}_{3}^{-}$ concentration in the surrounding seawater. In total, three batches of free-living *S. marinoi* cells and four batches of aggregate-associated *S. marinoi* cells were investigated at different extracellular ${NO}_{3}^{-}$ concentrations.

Free-living *S. marinoi* cells were cultivated in F/2 growth medium as described above, washed in nitrate-free NaCl solution (salinity 30), and then adjusted to 0, 10, 15, 25, 50 (*n* = 2), 60, 100 (*n* = 2), and 500 μmol L^-1^ ${NO}_{3}^{-}$ for 12–24 h, after which the intracellular ${NO}_{3}^{-}$ content of the diatom cells was measured in up to three replicates as described above.

For the production of diatom-bacteria aggregates, 50 mL of a stationary-phase *S. marinoi* culture was mixed with 550 mL natural Baltic Sea water (salinity adjusted to 30) and filled into four glass bottles, and sealed without bubbles. These aggregate-production bottles were mounted on a plankton wheel (diameter: 60 cm) and continuously rotated to induce aggregate formation and to always keep the aggregates sinking (Stief et al., 2016). After aggregates had formed (2–3 days), the water in the four aggregate-production bottles was adjusted to 15, 75, 100, or 350 μmol L^-1^ ${NO}_{3}^{-}$ and the bottles were rotated on the plankton wheel for another 24 h. The aggregates were then individually harvested from the aggregate-production bottles with a glass tube, sized along the three axes with a ruler, and transferred into a centrifugation tube together with water from the aggregate-production bottle still adhering to the aggregate. After the aggregate had settled, 100 μL of the supernatant was sampled and immediately frozen at -20°C until ${NO}_{3}^{-}$ analysis (see above). The remaining water was carefully removed without destroying the aggregate. The centrifugation tubes were vigorously mixed to arrive at a homogenous suspension. A subsample of the aggregate suspension was taken for diatom cell counts (see above), and the remaining aggregate suspension was frozen in liquid nitrogen, exposed to three freeze-thaw cycles to extract intracellular ${NO}_{3}^{-}$ (Heisterkamp et al., 2012), and stored at -20°C until ${NO}_{3}^{-}$ analysis (see above). The intracellular ${NO}_{3}^{-}$ concentration in the aggregate-associated diatom cells was calculated from the ${NO}_{3}^{-}$ concentrations in the supernatant and the extracted aggregate suspension, the diatom cell counts, and the aggregate volume (i.e., 3.5–6 mm^3^ for the four batches). The average cell volume of *S. marinoi* of 0.33 pL was taken from Kamp et al. (2011).

### Consumption of Intracellular Nitrate in Diatom-Bacteria Aggregates

Diatom-bacteria aggregates formed under oxic conditions in a light:dark cycle were investigated for the consumption of intracellular ${NO}_{3}^{-}$ after sudden shifts to dark and anoxic conditions, thereby mimicking conditions for an aggregate sinking through oxygen depleted waters.

Aggregates were produced in two separate batches (‘batch 1’ and ‘batch 2’) as described above and under light and oxic conditions, thereby mimicking aggregate formation and intracellular ${NO}_{3}^{-}$ accumulation in the euphotic zone. Aggregates of ‘batch 1’ were pre-incubated in Baltic Sea water (salinity adjusted to 30, ^14^${NO}_{3}^{-}$ adjusted to 75 μmol L^-1^) for 24 h. For the experimental incubation, seawater was flushed with N_2_ to remove O_2_ and filled into 6-mL incubation vials (Labco, UK). Aggregates of ‘batch 2’ were pre-incubated in 100 μmol L^-1^ ^15^${NO}_{3}^{-}$ for 12 h (see next section). For the experimental incubation, seawater was flushed with He to remove O_2_ and to lower the N_2_ background and filled into 6-mL incubation vials. Ammonium and acetate were not added because organic matter mineralization inside the aggregates was expected to cover the possible demands for N-assimilation and C-dissimilation by diatoms and bacteria. Single aggregates were harvested from the pre-incubation bottles, sized as described above, and transferred into the incubation vials. Incubation vials were sealed, wrapped in aluminum foil, and mounted on the plankton wheel (except for the t_0_ samples) to keep the aggregates sinking. At time intervals of 0, 3, 6, 24, and 48 h, four incubation vials each were sacrificed and the samples were processed as described above for ${NO}_{3}^{-}$ analysis in aggregate-associated diatom cells and whole aggregates.

### Anaerobic Turnover of Intracellular Nitrate in Diatom-Bacteria Aggregates

The conversion of intracellular vs. extracellular ${NO}_{3}^{-}$ to intermediates and products of dissimilatory ${NO}_{3}^{-}$ reduction inside sinking diatom-bacteria aggregates was investigated with a ^15^N-stable isotope experiment.

Diatom-bacteria aggregates were produced as described above in natural Baltic Sea water (salinity adjusted to 30), but without adding ^14^${NO}_{3}^{-}$ above the natural background concentration of 15 μmol ${NO}_{3}^{-}$ L^-1^. Instead, 100 μmol L^-1^ ^15^${NO}_{3}^{-}$ was added to the aggregate-production bottle 24 h prior to the experimental incubation to allow diatoms to accumulate ^15^${NO}_{3}^{-}$ intracellularly. Ellipsoidal aggregates of 6.1 ± 2.1 mm^3^ formed within 3 days (aggregate ‘batch 2’). Aggregates of this batch were also analyzed together with the aggregates of ‘batch 1’ with respect to storage and consumption of intracellular ${NO}_{3}^{-}$ (see previous section).

To remove all extracellular ^15^${NO}_{3}^{-}$ and to make intracellular ^15^${NO}_{3}^{-}$ the sole ^15^${NO}_{3}^{-}$ source in the experiment, aggregates were carefully washed in nitrate-free NaCl solution (salinity 30). After washing, the aggregates were sized with a ruler, and transferred into 6-mL incubation vials that were previously filled with anoxic Baltic Sea water (salinity adjusted to 30). Ammonium and acetate were not added for reasons given in the previous section. The incubation vials were sealed, wrapped in aluminum foil, and mounted on the rotating plankton wheel (except for the t_0_ samples) to keep the aggregates sinking.

(a) At time intervals of 0, 3, 6, 24, 48, and 72 h (diatom-free) water and intact aggregates were taken from three incubation vials each, frozen in liquid nitrogen to stop all metabolic activities and stored at -20°C until analysis of intracellular ^15^${NO}_{3}^{-}$ and extracellular ${NO}_{3}^{-}$, ${NH}_{4}^{+}$, and ${NO}_{2}^{-}$ (see above).

(b) At parallel time intervals of 3, 6, 24, and 72 h, a He headspace of 2.5 mL was set in three additional incubation vials each (note that this was done in additional incubation vials because setting the headspace tends to destroy the aggregates). The remaining sample volume of 3.5 mL was amended with 100 μL ZnCl_2_ (50% w/v). The incubation vials were stored upside-down at room temperature until measurement of ^15^N_2_ and N_2_O concentrations (see above). The cell suspensions that were collected during setting the headspace were centrifuged (5 min; 600 *g*) and the supernatants were used for measuring extracellular ^15^${NH}_{4}^{+}$ and ^15^${NO}_{2}^{-}$ concentrations.

The labeling fraction of the initial intracellular ${NO}_{3}^{-}$ pool of the diatoms was determined as described above and in this case was 37.2 ± 9.3% (*n* = 3). Assuming that this labeling fraction remains constant during the incubation (see above), the measured ^15^N-concentrations of products (i.e., ^15^${NO}_{2}^{-}$, ^15^${NH}_{4}^{+}$, and ^15^N_2_) were converted to intracellular ${NO}_{3}^{-}$-derived N-concentrations (i.e., ^IC^${NO}_{2}^{-}$, ^IC^${NH}_{4}^{+}$, and ^IC^N_2_) by dividing the ^15^N-concentrations by 0.372. Extracellular, ${NO}_{3}^{-}$-derived N-concentrations of ${NO}_{2}^{-}$ and ${NH}_{4}^{+}$ (i.e., ^EC^${NO}_{2}^{-}$ and ^EC^${NH}_{4}^{+}$) were calculated from the changes in measured total concentrations (i.e., ^TOT^${NO}_{2}^{-}$ and ^TOT^${NH}_{4}^{+}$) minus the calculated ^IC^N-concentrations. ^TOT^N_2_-concentrations were derived by using the principles of random isotope pairing, i.e., ^TOT^N_2_ = (^29^N_2_)^2^/(4 × ^30^N_2_) + ^29^N_2_ + ^30^N_2_ (Nielsen, 1992); and ^EC^N_2_-concentrations were calculated as described for ^EC^${NO}_{2}^{-}$ and ^EC^${NH}_{4}^{+}$. Application of the isotope pairing technique is based on the assumptions that (a) denitrification is the only significant N_2_-producing process in diatom aggregates (Stief et al., 2016) and (b) ^15^${NO}_{3}^{-}$ and ^14^${NO}_{3}^{-}$ are uniformly mixed at the site where denitrification takes place. The latter may not hold in the aggregates, if ^15^${NO}_{3}^{-}$ is uniformly released in the anoxic center, while ^14^${NO}_{3}^{-}$ decreases along a radial gradient into the center. This would result in an underestimation of ^TOT^N_2_ and thereby of ^EC^N_2_ concentrations.

## Results

### Dissimilatory Nitrate Reduction to Ammonium (DNRA) by Axenic *Skeletonema marinoi*

**FIGURE 1:**
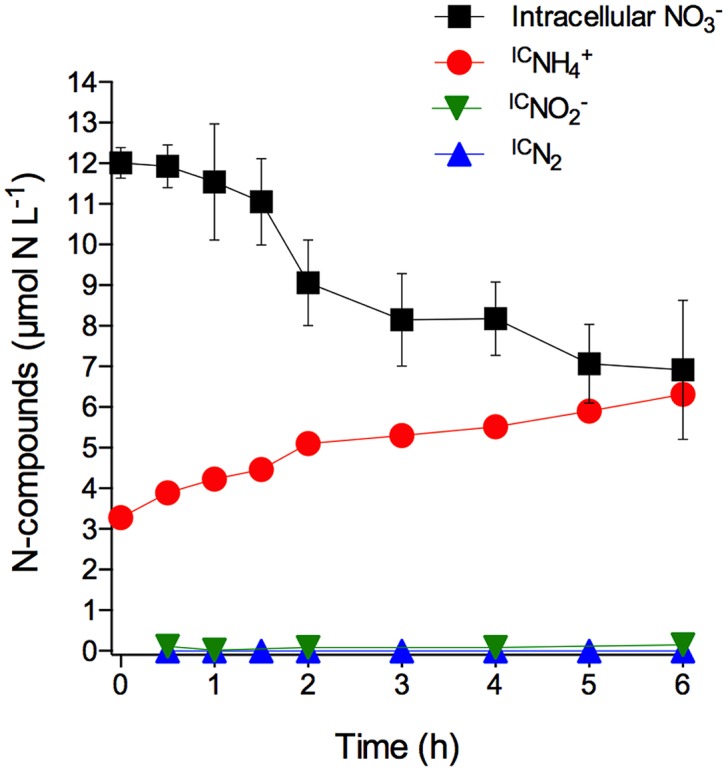
**Dissimilatory nitrate reduction to ammonium (DNRA) by axenic *Skeletonema marinoi*.** Time course of intracellular ${NO}_{3}^{-}$ (expressed in µmol NL^-1^ of growth medium; the initial ${NO}_{3}^{-}$ concentration *per cell* was 13.9 ± 0.4 mmol L^-1^) and extracellular, but intracellular ${NO}_{3}^{-}$ -derived ^IC^${NH}_{4}^{+}$, ^IC^${NO}_{2}^{-}$, and ^IC^N_2_ concentrations in axenic *S. marinoi* cultures in response to dark and anoxic conditions that were initiated directly after t_0_. Some of the error bars, which indicate standard deviation (*n* = 3), are smaller than the symbols.

The *S. marinoi* cells used in this experiment had stored intracellular ${NO}_{3}^{-}$ at a concentration of 13.9 ± 0.4 mmol L^-1^ (mean ± SD, *n* = 3). The time course of total intracellular ${NO}_{3}^{-}$ and intracellular ${NO}_{3}^{-}$-derived ^IC^${NO}_{2}^{-}$, ^IC^${NH}_{4}^{+}$, and ^IC^N_2_ concentrations in axenic *S. marinoi* cultures after the sudden shift to dark and anoxic conditions revealed that ${NH}_{4}^{+}$ was the only product arising from intracellular ${NO}_{3}^{-}$ consumption (**Figure 1**; **Supplementary Table S1**). A time-integrated mass balance calculation revealed that 90% of the intracellular ${NO}_{3}^{-}$ was converted to ^IC^${NH}_{4}^{+}$ during the incubation period of 6 h. The initial ^IC^${NH}_{4}^{+}$ concentration of 3.3 μmol L^-1^ is likely due to the production and release of ^IC^${NH}_{4}^{+}$ during the time period needed to shift the diatom culture to dark and anoxic conditions. Taken together, the data shows that *S. marinoi* is able to perform DNRA under dark and anoxic conditions (but see restrictive interpretation in the Introduction). Furthermore, the temporal coincidence of intracellular ${NO}_{3}^{-}$ consumption and ^IC^${NH}_{4}^{+}$ production rules out the possibility of ${NO}_{3}^{-}$ assimilation, followed by protein degradation and subsequent ${NH}_{4}^{+}$ release. Since ^IC^${NO}_{2}^{-}$ and ^IC^N_2_ production were not observed, *S. marinoi* seems incapable of dissimilatory ${NO}_{3}^{-}$ reduction to ${NO}_{2}^{-}$ (DNRN) as a stand-alone process, denitrification, and anammox.

Under light/oxic conditions, the intracellular ${NO}_{3}^{-}$ concentration decreased only slightly during the 6 h incubation (-0.06 ± 0.03 fmol ${NO}_{3}^{-}$ cell^-1^ h^-1^ under light/oxic conditions vs. -0.36 ± 0.04 fmol ${NO}_{3}^{-}$ cell^-1^ h^-1^ under dark/anoxic conditions; linear regression), probably due to assimilation and/or leakage of intracellular ${NO}_{3}^{-}$. In contrast to anoxic conditions, ${NH}_{4}^{+}$ was not released in measurable quantities by *S. marinoi* in oxic conditions (data not shown). Non-labeled ${NH}_{4}^{+}$ that was adjusted to 100 μmol L^-1^ in the growth medium prior to the experiment, was not taken up under anoxic conditions, but decreased under oxic conditions from 96 ± 14 to 43 ± 4 μmol ${NH}_{4}^{+}$ L^-1^ during the 6 h incubation, probably due to assimilation (data not shown).

### Intracellular Nitrate Storage by Free-Living and Aggregate-Associated *S. marinoi*

**FIGURE 2:**
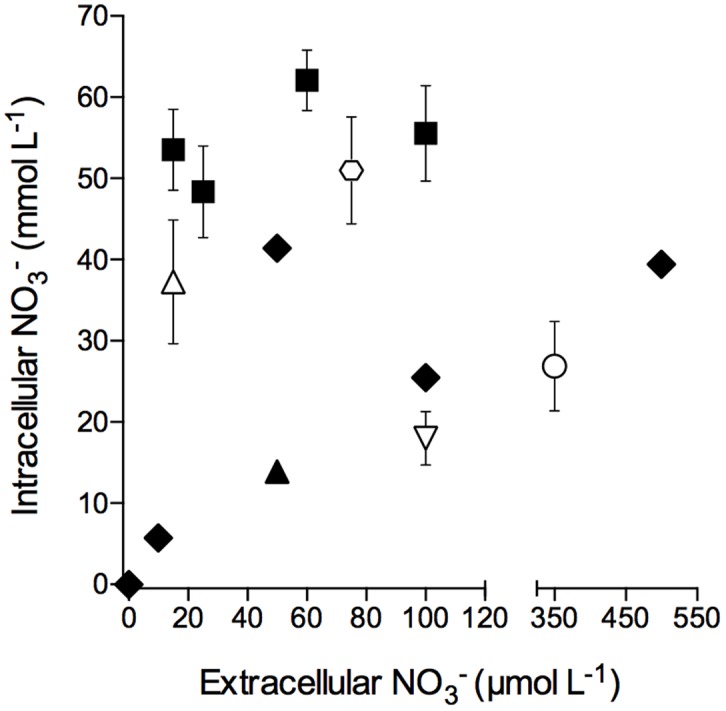
**Intracellular nitrate concentration of free-living and aggregate-associated *Skeletonema marinoi* cells at different extracellular nitrate concentrations.** Intracellular nitrate concentrations of free-living *S. marinoi* cells (black symbols) and aggregate-associated *S. marinoi* cells (white symbols) are expressed per cell volume; different symbol types show different batches. Extracellular ${NO}_{3}^{-}$ concentrations were adjusted 12–24 h prior to cell and aggregate sampling, respectively. Data points represent single measurements (black diamonds) or mean ± SD (*n* ≤ 14).

Intracellular ${NO}_{3}^{-}$ concentrations in free-living *S. marinoi* cells as well as in *S. marinoi* cells in diatom-bacteria aggregates grown or kept under light/oxic conditions were not correlated to extracellular ${NO}_{3}^{-}$ concentrations, with the exception that no intracellular ${NO}_{3}^{-}$ was detected at an extracellular ${NO}_{3}^{-}$ concentration of 0 μmol L^-1^ (**Figure 2**). Additionally, the ${NO}_{3}^{-}$ storage capacity of free-living and aggregate-associated *S. marinoi* cells was in the same concentration range (i.e., 6–62 and 18–51 mmol L^-1^, respectively).

### Consumption of Intracellular Nitrate in Diatom-Bacteria Aggregates

**FIGURE 3:**
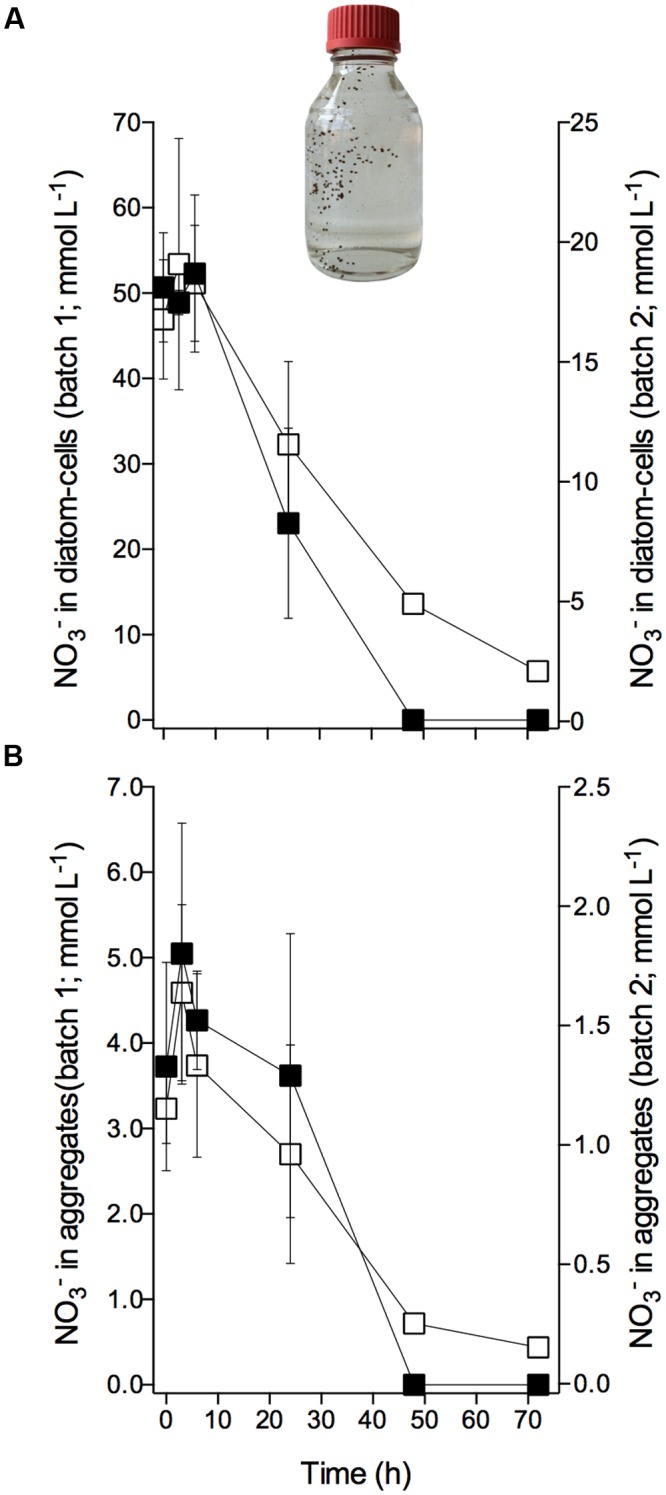
**Consumption of intracellular nitrate by *Skeletonema marinoi* in diatom-bacteria aggregates.** Time course of intracellular ${NO}_{3}^{-}$ concentrations in diatom-bacteria aggregates expressed per **(A)** diatom cell volume and **(B)** aggregate volume. **(A,B)** Left y-axes scale cells and aggregates of ‘batch 1’ (black squares), and right y-axes scale cells and aggregates of ‘batch 2’ (white squares). Dark and anoxic conditions were initiated directly after t_0_. Values are mean ± SD (*n* = 4 for ‘batch 1,’ *n* = 3 for ‘batch 2’). Picture shows an example of an aggregate-production bottle with sinking, brownish, ellipsoidal diatom-bacteria aggregates. Aggregate volumes were 4.1 ± 1.9 for ‘batch 1’ and 6.1 ± 2.1 mm^3^ for ‘batch 2’ (mean ± SD).

The diatom-bacteria aggregates had accumulated ${NO}_{3}^{-}$ at concentrations that were 2–3 orders of magnitude higher than extracellular ${NO}_{3}^{-}$ concentrations. In aggregate ‘batch 1,’ intracellular ${NO}_{3}^{-}$ was initially stored at concentrations of 51.0 ± 6.6 mmol L^-1^ based on diatom cell volume, and 4.2 ± 1.1 mmol L^-1^ based on aggregate volume [mean ± SD of 12 samples taken at 0–6 h (see below); **Figures 3A,B**]; in aggregate ‘batch 2,’ initial intracellular ${NO}_{3}^{-}$ was 18.0 ± 3.3 mmol L^-1^ per cell, and 1.4 ± 0.3 mmol L^-1^ per aggregate (mean ± SD of nine samples taken at 0–6 h; **Figures 3A,B**). Assuming that the extracellular ${NO}_{3}^{-}$ concentration in the porewater of the aggregate was similar to the adjusted extracellular ${NO}_{3}^{-}$ concentration (i.e., 75 and 100 μmol L^-1^ in ‘batch 1’ and ‘batch 2,’ respectively), the standing stock of ${NO}_{3}^{-}$ inside diatom-bacteria aggregates was largely (i.e., ≥98%) contained in diatom cells.

Within the first 6 h of incubation under dark and anoxic conditions, the intracellular ${NO}_{3}^{-}$ concentration of the diatom-bacteria aggregates did not change significantly over time (**Figures 3A,B**; **Supplementary Table S2**). The scatter in the data may, however, have masked a potential decrease in intracellular ${NO}_{3}^{-}$ concentration during this short time period. After 6 h, however, intracellular ${NO}_{3}^{-}$ was rapidly consumed in aggregate ‘batch 1’ at a rate of -0.37 ± 0.03 fmol ${NO}_{3}^{-}$ diatom-cell^-1^ h^-1^ or -361.78 ± 95.12 nmol ${NO}_{3}^{-}$ aggregate^-1^ h^-1^. The intracellular pool was completely consumed after 48 h. In aggregate ‘batch 2,’ intracellular ${NO}_{3}^{-}$ consumption within the first 48 h was -0.09 ± 0.01 fmol ${NO}_{3}^{-}$ diatom-cell^-1^ h^-1^ or -145.00 ± 35.79 nmol ${NO}_{3}^{-}$ aggregate^-1^ h^-1^, but continued at a lower rate after 48 h and ${NO}_{3}^{-}$ was not completely used up after 72 h (**Figures 3A,B**; **Supplementary Table S2**). Thus, irrespective of the different conditions that the aggregates experienced during the pre-incubation (regarding extracellular ${NO}_{3}^{-}$ concentration and exposure time), the aggregates displayed the same temporal pattern of intracellular ${NO}_{3}^{-}$ consumption during incubation under dark and anoxic conditions.

### Anaerobic Turnover of Intracellular Nitrate in Diatom-Bacteria Aggregates

**FIGURE 4:**
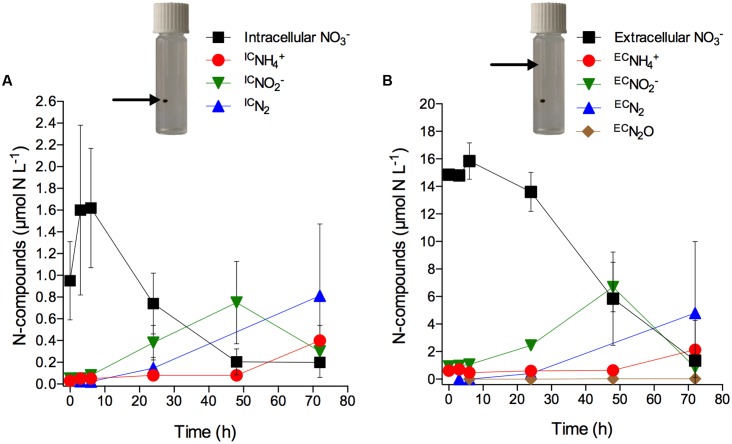
**Anaerobic turnover of intracellular nitrate in diatom-bacteria aggregates.** Concentration time series of **(A)** intracellular ${NO}_{3}^{-}$ (expressed in μmol N L^-1^ of incubation water; initial ${NO}_{3}^{-}$ concentration *per cell* was 18.0 ± 3.3 mmol L^-1^) and intracellular ${NO}_{3}^{-}$-derived ^IC^${NH}_{4}^{+}$, ^IC^${NO}_{2}^{-}$, and ^IC^N_2_, and **(B)** extracellular ${NO}_{3}^{-}$ and extracellular ${NO}_{3}^{-}$-derived ^EC^${NH}_{4}^{+}$, ^EC^${NO}_{2}^{-}$, ^EC^N_2_, and ^EC^N_2_O (see Materials and Methods for the calculation of ^IC^N- and ^EC^N-concentrations). Dark and anoxic conditions were initiated directly after t_0_. Values are means ± SD (*n* = 3). Note different scales. Pictures show incubation vials with diatom-bacteria aggregates; arrows indicate the respective source of ${NO}_{3}^{-}$ that drives anaerobic nitrogen cycling (i.e., intracellular ${NO}_{3}^{-}$ inside the aggregate vs. extracellular ${NO}_{3}^{-}$ in the surrounding water).

Only after 6 h of incubation, significant concentration changes of intra- and extracellular ${NO}_{3}^{-}$ as well as of extracellular ${NO}_{2}^{-}$, N_2_, and ${NH}_{4}^{+}$ were observed (**Figures 4A,B**; **Supplementary Table S3**). What may look like an increase in intracellular ${NO}_{3}^{-}$ concentration within the first 6 h of incubation is actually not statistically significant (**Figure 4A**; **Supplementary Table S3**). However, scatter in the data may have masked a potential net turnover of intra- and extracellular ${NO}_{3}^{-}$ during this initial time period. In contrast to the exclusive production of ${NH}_{4}^{+}$ in the axenic cultures, the consumption of intracellular ${NO}_{3}^{-}$ during anoxic incubation of aggregates was accompanied by the production and release of ^IC^${NO}_{2}^{-}$, ^IC^N_2_, and ^IC^${NH}_{4}^{+}$ from the aggregates (**Figure 4A**). Extracellular ${NO}_{3}^{-}$ present in the seawater showed a similar temporal pattern of consumption and was likewise accompanied by the production and release of ^EC^${NO}_{2}^{-}$, ^EC^N_2_, and ^EC^${NH}_{4}^{+}$ from the aggregates, though at higher concentrations than observed for the intracellular ${NO}_{3}^{-}$-derived products (**Figure 4B**). This likely reflects the higher supply rate of extracellular ${NO}_{3}^{-}$ diffusing into the aggregate from the surrounding water compared to the presumably slow release of intracellular ${NO}_{3}^{-}$ from the diatom cells into the aggregate. Both ^IC^${NO}_{2}^{-}$ and ^EC^${NO}_{2}^{-}$ concentrations peaked after 48 h incubation and decreased thereafter. In contrast, ^IC^${NH}_{4}^{+}$ and ^EC^${NH}_{4}^{+}$ concentrations increased significantly only after 48 h. ^IC^N_2_ and ^EC^N_2_ were produced from the onset and throughout the entire incubation period. A time-integrated budget shows that in total 95% of the intracellular ${NO}_{3}^{-}$ was consumed within the first 48 h and retrieved as ^IC^${NO}_{2}^{-}$ (59%), ^IC^N_2_ (31%), and ^IC^${NH}_{4}^{+}$ (5%). In contrast, only 49% of the extracellular ${NO}_{3}^{-}$ that was consumed within the first 48 h was retrieved as ^EC^${NO}_{2}^{-}$ (35%), ^EC^N_2_ (14%), and ^EC^${NH}_{4}^{+}$ (<1%).

Notably, in the diatom-bacteria aggregates, the production of ^IC^${NH}_{4}^{+}$ did not coincide with the consumption of intracellular ${NO}_{3}^{-}$, as was the case in the experiments with axenic *S. marinoi* (**Figure 1**; **Supplementary Table S1**), but occurred with a delay of ca. 2 days (**Figures 4A,B**; **Supplementary Table S3**). Additionally, ^IC^${NO}_{2}^{-}$ and ^IC^N_2_ were important products of intracellular ${NO}_{3}^{-}$ consumption in the diatom-bacteria aggregates, but not in the axenic *S. marinoi* cultures. Thus, intracellular ${NO}_{3}^{-}$ must have been made accessible to other microorganisms inside the diatom-bacteria aggregates contributing to complex anaerobic nitrogen cycling during the dark and anoxic incubation.

## Discussion

### Dissimilatory Nitrate Reduction to Ammonium (DNRA) by Axenic *Skeletonema marinoi*

The aggregate-forming diatom *S. marinoi* uses intracellularly stored ${NO}_{3}^{-}$ for the dissimilatory ${NO}_{3}^{-}$ reduction pathway DNRA under dark and anoxic conditions and is thus the third diatom species that has been positively tested for DNRA in axenic cultures. Previously, DNRA has been found in the benthic diatom *A. coffeaeformis* and the pelagic, aggregate-forming *T. weissflogii* (Kamp et al., 2011, 2013, 2015). Thus, DNRA might be a widely distributed anaerobic metabolic pathway used by diatoms that are (temporarily) exposed to anoxic conditions in the dark, where neither photosynthesis, nor aerobic respiration is possible.

Dissimilatory nitrate reduction to ammonium activity by *S. marinoi* is fueled by intracellular ${NO}_{3}^{-}$ that has been accumulated under light and oxic conditions, where it is used for assimilation (e.g., Eppley and Rogers, 1970; Clark et al., 2002; Brown et al., 2009; Bender et al., 2012; Glibert et al., 2016) or stored for later use in assimilatory or dissimilatory pathways. Nitrate uptake under anoxic conditions has, to our knowledge, not been documented and was also not observed in this study (**Supplementary Figure S1**). The maximum intracellular ${NO}_{3}^{-}$ concentration found in *S. marinoi* was 62.0 ± 0.7 mmol L^-1^ and was measured in cells that had been exposed to an extracellular ${NO}_{3}^{-}$ concentration of 58 μmol L^-1^. Intracellular and extracellular ${NO}_{3}^{-}$ concentrations were apparently not directly correlated for *S. marinoi*, at least not in the targeted concentration range (**Figure 2**). The ${NO}_{3}^{-}$ uptake and/or storage capacity of *S. marinoi* might depend on factors other than the extracellular NO_3_ concentration, such as temperature (Eppley and Coatsworth, 1968; Collos et al., 1992; Lomas and Glibert, 1999a; Tantanasarit et al., 2013), the physiological state of the cells, or the extracellular ${NH}_{4}^{+}$ concentration (Lomas and Glibert, 1999b; Glibert et al., 2016).

Interestingly, the depletion of the intracellular ${NO}_{3}^{-}$ pool of *S. marinoi* under anoxic conditions may take more than 2 days (**Figure 3**), whereas *A. coffeaeformis* and *T. weissflogii* completely use up their intracellular ${NO}_{3}^{-}$ pools within less than 24 h after exposure to darkness and anoxia (Kamp et al., 2011, 2013). This was supported by the observation that *S. marinoi* cells that were first subjected to the “starvation procedure” and then exposed to ^15^${NO}_{3}^{-}$ had an isotopically mixed intracellular ${NO}_{3}^{-}$ pool (i.e., ^14^${NO}_{3}^{-}$ and ^15^${NO}_{3}^{-}$). The intracellular ${NO}_{3}^{-}$ consumption rate immediately after exposure of *S. marinoi* to darkness and anoxia was 3–25 times lower for *S. marinoi* (0.36 fmol ${NO}_{3}^{-}$ cell^-1^ h^-1^, **Figure 1**) than for *T. weissflogii* (1.15–7.47 fmol ${NO}_{3}^{-}$ cell^-1^ h^-1^; Kamp et al., 2013), and *A. coffeaeformis* (9.1 fmol ${NO}_{3}^{-}$ cell^-1^ h^-1^; Kamp et al., 2011). Likewise, the absolute amount of ${NO}_{3}^{-}$ stored per cell was 7–35 times lower for *S. marinoi* (1.9–20.4 fmol ${NO}_{3}^{-}$ cell^-1^, **Figure 2**) than for *T. weissflogii* (113 fmol ${NO}_{3}^{-}$ cell^-1^; Kamp et al., 2011) and *A. coffeaeformis* (129 fmol ${NO}_{3}^{-}$ cell^-1^; Kamp et al., 2011). Thus, despite the roughly similar ratio between pool size and consumption rate of intracellular ${NO}_{3}^{-}$ among the three investigated diatom species, the intracellular ${NO}_{3}^{-}$ pool in *S. marinoi* can sustain DNRA for a longer period than *T. weissflogii* and *A. coffeaeformis*. The intracellular ${NO}_{3}^{-}$ consumption rate of *S. marinoi* depends, however, on the initial intracellular ${NO}_{3}^{-}$ concentration. The more ${NO}_{3}^{-}$ that is stored, the higher the consumption rate because ${NO}_{3}^{-}$ is largely consumed within a certain time frame (approximately 1–2 days *for S. marinoi*). The consumption rate might actually be overestimated, if ${NO}_{3}^{-}$ leaks out of the cell, e.g., if the high concentration gradient between intra- and extracellular ${NO}_{3}^{-}$ is not maintainable in darkness and anoxia. Nitrate leakage may also partly explain that only 90% of the intracellular ${NO}_{3}^{-}$ lost during the 6-h incubation was retrieved as ${NH}_{4}^{+}$.

Intracellular ${NO}_{3}^{-}$ consumption rates of foraminifera that are capable of denitrification in the absence of O_2_ are ∼100 times higher (1.7–83 pmol cell^-1^ h^-1^; Risgaard-Petersen et al., 2006; Høgslund et al., 2008; Piña-Ochoa et al., 2010a,b; Bernhard et al., 2012) than those of diatoms capable of DNRA, while the absolute amounts of ${NO}_{3}^{-}$ stored per cell are up to ∼10^5^ times higher (18 nmol ${NO}_{3}^{-}$ cell^-1^; Risgaard-Petersen et al., 2006). Therefore, it is not surprising that the intracellular ${NO}_{3}^{-}$ stores of foraminifera were estimated to sustain denitrification and thus survival under anoxic conditions for much longer periods (i.e., over a month; Risgaard-Petersen et al., 2006; Glud et al., 2009). In contrast, it was hypothesized that diatoms use intracellular ${NO}_{3}^{-}$-fueled DNRA for entering a resting stage rather than for long-term survival with an active anaerobic metabolism (Kamp et al., 2011). Diatoms are generally known to survive dark and anoxic conditions in marine sediments for several years or even decades as resting spores (Lewis et al., 1999; McQuoid et al., 2002; Jewson et al., 2006; Härnström et al., 2011). Given the large phylogenetic diversity of diatoms of up to 100.000 species (Leblanc et al., 2012) and the pronounced genetic variation in diatoms (Armbrust et al., 2004; Bowler et al., 2008; Prihoda et al., 2012), it seems likely that diatoms have also evolved mechanisms other than DNRA to remain metabolically active during darkness and anoxia, or hitherto unrecognized pathways of anaerobic metabolism.

### Anaerobic Turnover of Intracellular Nitrate in Diatom-Bacteria Aggregates

Diatom-bacteria aggregates produced from axenic *S. marinoi* and the natural bacterial community of coastal seawater contained high amounts of intracellular ${NO}_{3}^{-}$ at the end of the aggregate-production phase in a diel light:dark cycle. It has previously been shown for *S. marinoi* aggregates that the total intracellular ${NO}_{3}^{-}$ content increases with aggregate volume, whereas the aggregate-volume-specific intracellular ${NO}_{3}^{-}$ content decreases with aggregate volume (Stief et al., 2016). The first observation is consistent with this study, with a larger number of diatom cells in larger aggregates that can store more intracellular ${NO}_{3}^{-}$ in absolute amounts (**Supplementary Figure S2**). Aggregates larger than 2 μL in volume had a lower diatom cell density than the smaller aggregates, which is consistent with the previously observed lower volume-specific intracellular ${NO}_{3}^{-}$ content of larger aggregates. This may indicate that the diatom cells in the outer shell of the aggregates mainly take up ${NO}_{3}^{-}$ from the surrounding water because ${NO}_{3}^{-}$ transport into the center of the aggregates is diffusion-limited. However, direct comparison of free-living and aggregate-associated *S. marinoi* cells did not reveal any difference in ${NO}_{3}^{-}$ storage capacity (6–63 and 18–51 mmol L^-1^, respectively, **Figure 2**), rendering a strong influence of diffusion limitation on ${NO}_{3}^{-}$ transport unlikely. Additionally, the high cell densities in the aggregates and the possible competition for ${NO}_{3}^{-}$ do not seem to lower the ability to accumulate ${NO}_{3}^{-}$ in aggregate-associated *S. marinoi* cells.

When diatom-bacteria aggregates were exposed to dark and anoxic conditions, their intracellular ${NO}_{3}^{-}$ content was used up within 2–3 days at similar cellular rates as observed in the axenic, free-living *S. marinoi*. Based on the cell-specific intracellular ${NO}_{3}^{-}$ consumption rate of axenic *S. marinoi* (0.36 fmol ${NO}_{3}^{-}$ cell^-1^ h^-1^) and the total number of *S. marinoi* cells in aggregates (0.5–2.1 × 10^6^ cells aggregate^-1^), an intracellular ${NO}_{3}^{-}$ consumption rate of 0.18–0.76 nmol ${NO}_{3}^{-}$ aggregate^-1^ h^-1^ can be projected. This agrees reasonably well with the measured intracellular ${NO}_{3}^{-}$ consumption rates of 0.14–0.36 nmol ${NO}_{3}^{-}$ aggregate^-1^ h^-1^. In sharp contrast to axenic *S. marinoi* cultures, however, the consumption of intracellular ${NO}_{3}^{-}$ by aggregate-associated *S. marinoi* cells was not accompanied by the concurrent release of ${NH}_{4}^{+}$, which argues against DNRA by aggregate-associated *S. marinoi* as a major nitrate sink. Instead, the ^15^N-labeling experiment clearly indicated that much of the ${NO}_{3}^{-}$ initially stored by *S. marinoi* was used for dissimilatory ${NO}_{3}^{-}$ reduction by the diverse microbial community of the aggregates. This interpretation is supported by the observations that (a) only a small fraction of the intracellular ${NO}_{3}^{-}$ was converted to ^IC^${NH}_{4}^{+}$ (the pathway that can be carried out by axenic *S. marinoi*), while a much larger fraction was converted to ^IC^${NO}_{2}^{-}$ and ^IC^N_2_ (that were not produced by axenic *S. marinoi*), (b) the aggregates produced ^IC^${NH}_{4}^{+}$ with a delay of 2 days, whereas axenic *S. marinoi* produced ^IC^${NH}_{4}^{+}$ immediately after the onset of dark and anoxic conditions, and (c) the time course and the products of dissimilatory ${NO}_{3}^{-}$ reduction inside the aggregates were very similar, irrespective of whether driven by intracellular or extracellular ${NO}_{3}^{-}$.

The mechanism of intracellular ${NO}_{3}^{-}$ transfer from the diatom cells to the microbial community of the aggregates is currently unknown. Living diatom cells inside the aggregates may continuously leak ${NO}_{3}^{-}$ under anoxic conditions, especially if the aggregate porewater is ${NO}_{3}^{-}$-depleted and the diatoms are unable to maintain the steep concentration gradient across the plasma membrane. Under dark and anoxic conditions, diatoms are apparently unable to refill their intracellular ${NO}_{3}^{-}$ stores (see above), which would then lead to a net loss of intracellular ${NO}_{3}^{-}$. However, ${NO}_{3}^{-}$ leakage was never observed in axenic cultures of *S. marinoi*, nor in *T. weissflogii* or *A. coffeaeformis*, since the extracellular ${NO}_{3}^{-}$ concentration does not increase while the intracellular ${NO}_{3}^{-}$ is consumed under dark and anoxic conditions (Kamp et al., 2011, 2013). Alternatively, decaying or lysing diatom cells may gradually release intracellular ${NO}_{3}^{-}$ into the aggregate if, for instance, cell lysis is triggered by viral infection (Kimura and Tomarua, 2015; Kim et al., 2015). Viral-mediated mortality and cellular lysis could be especially high in the aggregates with high cell densities (Brussaard, 2004). *S. marinoi*, like many other microalgae, is also known to undergo programmed cell death (PCD) when stressed (Bidle and Falkowski, 2004; Orefice et al., 2015). Anoxia inside the aggregates might act as the stressor triggering PCD in diatoms.

An alternative scenario could be that aggregate-associated *S. marinoi* only reduce ${NO}_{3}^{-}$ to ${NO}_{2}^{-}$, and the ${NO}_{2}^{-}$ is then immediately excreted by the cell to be further reduced to N_2_ and ${NH}_{4}^{+}$ by the microbial community of the aggregates. *S. marinoi* is known to survive dark and anoxic conditions for much longer than the 6 and 72 h covered in the ^15^N-stable isotope experiments with axenic cultures and diatom-bacteria aggregates, respectively. Axenic *S. marinoi* was viable after both the 24-h starvation procedure in this study, and the 9-week incubation under dark and anoxic conditions in a previous study (Kamp et al., 2011). Thus, it cannot be ruled out that the aggregate-associated *S. marinoi* remained viable during the 72-h incubation under dark and anoxic conditions and displayed DNRN activity. It is currently not known, however, why *S. marinoi* should potentially shift from DNRA to DNRN when associated with sinking aggregates, but the observed increase in ^IC^${NO}_{2}^{-}$ concentration indeed coincides with the decrease in intracellular ${NO}_{3}^{-}$ concentration. Additionally, this scenario would explain both the similar rates and the gradual nature of intracellular ${NO}_{3}^{-}$ consumption in free-living vs. aggregate-associated *S. marinoi* (see above). Nitrite excretion is a common phenomenon in marine phytoplankton, including diatoms (Collos, 1998; Mackey et al., 2011) and has also been reported for an axenic strain of *T. weissflogii* capable of DNRA (Kamp et al., 2013).

### Ecological Implications of Intracellular Nitrate in Diatom-Bacteria Aggregates

Sinking aggregates mediate much of the vertical carbon export to the seafloor and thereby represent an integral component of the “biological pump” in the ocean (Turner, 2015). Our finding that diatom-bacteria aggregates store intracellular ${NO}_{3}^{-}$ in high amounts suggests that sinking aggregates may also be involved in the vertical transport of ${NO}_{3}^{-}$ to deep water layers or even to the seafloor. Nitrate accumulation by diatoms only occurs in the presence of O_2_ and thus diatom-bacteria aggregates may take up ${NO}_{3}^{-}$ in the euphotic zone and oxygenated subsurface layers and export it to the deep ocean.

Depending on the initial amount of ${NO}_{3}^{-}$ stored in sinking diatom-bacteria aggregates, the intracellular ${NO}_{3}^{-}$ consumption rate, the internal and ambient O_2_ concentration, temperature, the water depth, and the sinking velocity of the aggregates, a fraction of the intracellular ${NO}_{3}^{-}$ stores might reach the seafloor. At the experimental conditions of this study (i.e., 15°C, 0 μmol O_2_ L^-1^), the half-life of the intracellular ${NO}_{3}^{-}$ pool of the aggregates was ∼24 h. Sinking velocities of diatom-bacteria aggregates of the same size as used in this study are in the range of 50–300 m d^-1^ (Iversen and Ploug, 2013). Thus, settling aggregates that still contain half of their initial intracellular ${NO}_{3}^{-}$ content would be expected to settle on sediments at 50–300 m water depth where they may sustain benthic denitrification (Lehto et al., 2014). Aggregates exposed to lower temperatures and higher ambient O_2_ concentrations *in situ* could transport intracellular ${NO}_{3}^{-}$ down to considerably greater depths. More experimental work and modeling efforts will be necessary to refine these estimates. Irrespective of its unsettled quantification, the export of intracellular ${NO}_{3}^{-}$ to deep water layers in the ocean represents a rarely considered mechanism of fixed-nitrogen loss from the euphotic zone (Lehto et al., 2014; Stief et al., 2016). Future *in situ* studies should also quantify the transport of intracellular ${NO}_{3}^{-}$ by sinking aggregates in comparison to the successive depletion of their organic fraction in nitrogen relative to carbon (Martin et al., 1987; Smith et al., 1992; Dang and Lovell, 2016).

The intracellular ${NO}_{3}^{-}$ pool in diatom-bacteria aggregates may allow diatoms to survive anoxic conditions while the aggregates sink through oxygen-depleted water layers (Kamp et al., 2011). Pelagic diatoms are well-known to survive the descent to the seafloor even at great water depth (Fileman et al., 1998) and can indeed be found in viable resting stages in marine sediments (Lewis et al., 1999; Härnström et al., 2011). As shown in this study, the diatom-derived ${NO}_{3}^{-}$ also drives the metabolic activity of the (facultative) anaerobic bacterial community of the aggregates. The presence of bacteria actively mediating dissimilatory ${NO}_{3}^{-}$ reduction has been repeatedly confirmed for marine snow, small particles, and sinking zooplankton carcasses (Tuomainen et al., 2003; Woebken et al., 2007; Ganesh et al., 2014, 2015; Glud et al., 2015). The internal availability of ${NO}_{3}^{-}$ may not only allow these bacteria to remain active under anoxic conditions, but may also act as a selection factor in the succession of bacterial communities in sinking diatom-bacteria aggregates. Marine snow is mainly colonized by bacteria in the euphotic zone, but the microbial community composition is known to change during the descent (Tang et al., 2010; Thiele et al., 2015), which potentially is influenced by the presence of an internal ${NO}_{3}^{-}$ source when O_2_ is absent.

The ${NO}_{3}^{-}$ initially stored by diatom cells is expected to drive intense anaerobic nitrogen cycling inside partially or completely anoxic diatom-bacteria aggregates sinking to the seafloor. In this study, the rates of ${NO}_{2}^{-}$, N_2_, and ${NH}_{4}^{+}$ production driven by diatom-derived ${NO}_{3}^{-}$ were ∼7 times lower than those fueled by extracellular ${NO}_{3}^{-}$. This indicates that inside the aggregate the supply rate of intracellular ${NO}_{3}^{-}$ for anaerobic nitrogen cycling was lower than that of extracellular ${NO}_{3}^{-}$. However, the relative importance of intracellular ${NO}_{3}^{-}$ is expected to be higher at lower extracellular ${NO}_{3}^{-}$ concentrations and in larger, diffusion-limited aggregates (Stief et al., 2016). Notably, intracellular ${NO}_{3}^{-}$ is an internal ${NO}_{3}^{-}$ source that diatom-bacteria aggregates can exploit even in ${NO}_{3}^{-}$-depleted environments.

The finding that the diatom *S. marinoi* is capable of DNRA suggests that diatom-bacteria aggregates represent a pelagic ${NH}_{4}^{+}$ source in addition to organic matter mineralization (Kalvelage et al., 2013). The potential role of sinking aggregates and suspended particles as an important ${NH}_{4}^{+}$ source for the anammox process in OMZs has been discussed before (Dalsgaard et al., 2012; Kalvelage et al., 2013; Stief et al., 2016). Aggregate-associated DNRA activity may thereby fuel the anammox activity of free-living or particle-associated bacteria and thus be indirectly involved in fixed-nitrogen loss from the ocean (Stief et al., 2016). The results of this study, however, clearly indicate that at least part of the intracellular ${NO}_{3}^{-}$ stored by aggregate-associated diatoms drives fixed-nitrogen loss directly inside the aggregates. Intracellular ${NO}_{3}^{-}$ is transferred from the diatoms to the bacterial community of the aggregates, potentially aided by viral-induced cell lysis (Kim et al., 2015; Kimura and Tomarua, 2015), and then converted to nitrogen gas by denitrification. Toward the end of the 3-day incubation, significant ^IC^${NH}_{4}^{+}$ production was also observed, but since the intracellular ${NO}_{3}^{-}$ pool was depleted by that time, this ${NH}_{4}^{+}$ production was most likely mediated by bacteria.

**FIGURE 5:**
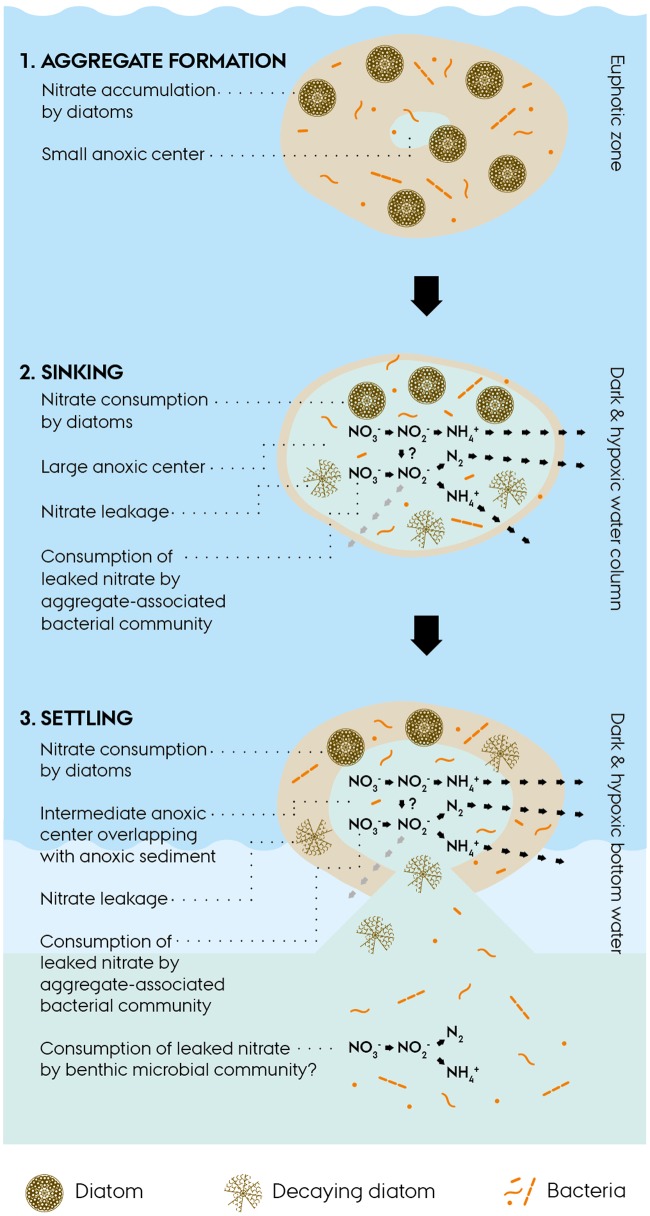
**Conceptual scheme of intracellular nitrate dynamics in diatom-bacteria aggregates.** (1) Diatom-bacteria aggregates form in the euphotic zone of the ocean. Aggregate-associated diatoms accumulate nitrate from the surrounding water and thereby up-concentrate nitrate within the aggregate. An anoxic center may develop within the aggregate, if oxygen consumption exceeds oxygen diffusion from the surrounding water, e.g., during the night when photosynthesis is not active. (2) Aggregates sink through dark and hypoxic water layers and develop a large anoxic center (Stief et al., 2016) or become completely anoxic, if sinking through oxygen-depleted water layers, which in both cases sustains anaerobic nitrogen cycling within the aggregates. Living diatoms consume their intracellular nitrate stores for dissimilation, whereas decaying diatoms leak intracellular nitrate into the aggregate and thereby make it available to the aggregate-associated bacterial community for complex anaerobic nitrogen cycling. (3) Aggregates settle onto the seafloor and their intracellular nitrate fuels benthic anaerobic nitrogen cycling in partly anoxic aggregates and sediment areas that turn anoxic due to the presence of the aggregates (Lehto et al., 2014).

The environmental scenario emerging from this study on intracellular ${NO}_{3}^{-}$ dynamics in diatom-bacteria aggregates can be crudely split into three consecutive phases (**Figure 5**):

(a) Under the light and oxic conditions in the euphotic zone, diatoms are able to accumulate ${NO}_{3}^{-}$ intracellularly, even against a steep concentration gradient. Due to the high ambient O_2_ levels, the anoxic center of aggregates will be small or even absent and hence the rates of anaerobic nitrogen cycling will be low or zero.

(b) During the descent, aggregates may pass through layers of reduced O_2_ levels, which will increase the anoxic volume inside the aggregate and sustain anaerobic nitrogen cycling. Living diatoms will perform DNRA, while decaying diatoms will pass on their intracellular ${NO}_{3}^{-}$ stores to the aggregate-associated bacterial community which performs diverse processes of anaerobic nitrogen cycling.

(c) Upon settlement of the aggregates onto the seafloor, the remaining intracellular ${NO}_{3}^{-}$ stores (if any) may fuel benthic anaerobic nitrogen cycling, which might be further stimulated by the induction of anoxic conditions in the sediment around the aggregate.

In summary, the nitrate-concentrating capacity of aggregate-associated diatoms has the potential to impact nitrogen cycling, including fixed-nitrogen loss, not only in the photic zone, but also in the mesopelagic and benthic compartments.

## Author Contributions

AK, PS, BT, and RG designed the study. AK and PS carried out the experiments. AK, PS, and LB measured the samples. All authors interpreted the data. AK wrote the manuscript with input from all co-authors.

## Conflict of Interest Statement

The authors declare that the research was conducted in the absence of any commercial or financial relationships that could be construed as a potential conflict of interest.
