# Supplementary material for: Intracellular Nitrate of Marine Diatoms as a Driver of Anaerobic Nitrogen Cycling in Sinking Aggregates
Source: Front Microbiol. 2016 Nov 1;7:1669. doi: 10.3389/fmicb.2016.01669 (PMC5088207; doi:10.3389/fmicb.2016.01669)
Supplement: Supplementary file 1 [file Data_Sheet_1.pdf]

Supplementary material

Intracellular Nitrate of Marine Diatoms as a Driver of Anaerobic Nitrogen Cycling in Sinking Aggregates

Anja Kamp, Peter Stief, Laura A. Bristow, Bo Thamdrup and Ronnie N. Glud

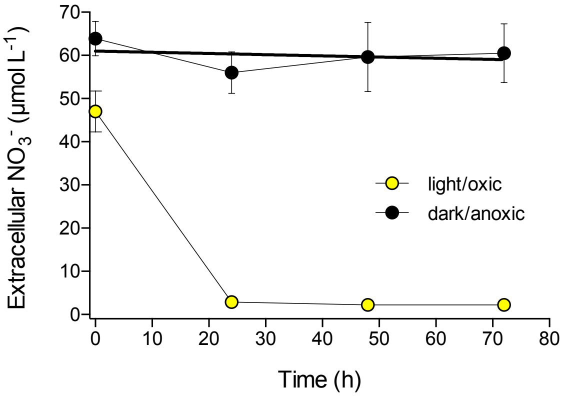

Figure S1 | Nitrate uptake of *Skeletonema marinoi* cells in light/oxic and dark/anoxic conditions. *S. marinoi* cells from a stationary-phase culture are taking up nitrate from the growth medium within one day in light/oxic conditions (n=6), but not in dark/anoxic conditions (n=6). The slope of the regression line of nitrate uptake in dark/anoxic conditions is not significantly different from zero (p=0.74). Error bars are sometimes within the symbol.

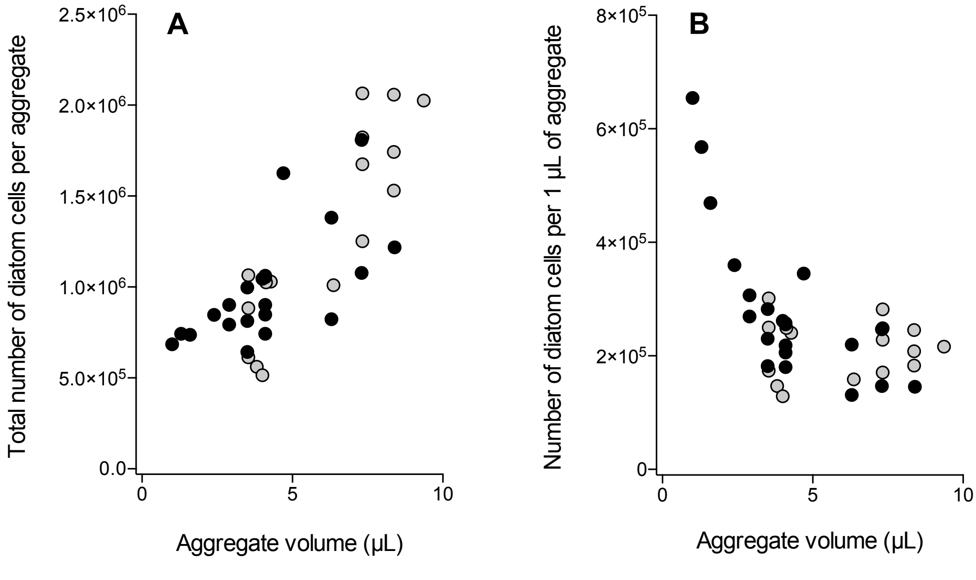

Figure S2 | Correlation between number of *Skeletonema marinoi* cells and aggregate volume. (A) Total number of diatom cells per aggregate and (B) number of diatom cells per µL of aggregate. Data were compiled from all aggregates of 'batch 1' (black) and 'batch 2' (grey).

Table S1 | Non-parametric correlation analysis of N-turnover versus time for incubations of axenic *S. marinoi* (Fig. 1).

	$ICNO_3$	NO_2^-	NH_4^+	N_2
Time	$R = –0.875$ $p < 0.01$	$R = 0.300$ $p = 0.62$	$R = 0.989$ $p < 0.01$	$R = –0.315$ $p = 0.13$

Positive and negative Spearman’s rank coefficients *R* indicate net production and consumption of N-compounds, respectively.
Significance levels $p < 0.05$ indicate that the net turnover of $ICNO_3$ is significantly different from zero.
Significant correlations are marked by a red frame.
$ICNO_3$ = intracellular nitrate

Table S2 | Non-parametric correlation analysis of $ICNO_3$-consumption versus time for incubations of diatom-bacteria aggregates prepared in two separate batches with *S. marinoi* (Fig. 3).

	$ICNO_3$ per cell		$ICNO_3$ per aggregate	
Time interval	Batch 1	Batch 2	Batch 1	Batch 2
0-6 h	$R = 0.234$ $p = 0.52$	$R = 0.264$ $p = 0.49$	$R = 0.160$ $p = 0.68$	$R = 0.369$ $p = 0.33$
6-48 h	$R = –0.933$ $p < 0.01$	$R = –0.945$ $p < 0.01$	$R = –0.759$ $p = 0.01$	$R = –0.869$ $p = 0.01$

Positive and negative Spearman’s rank coefficients *R* indicate net production and consumption of $ICNO_3$, respectively.
Significance levels $p < 0.05$ indicate that the net turnover of $ICNO_3$ is significantly different from zero.
Significant correlations are marked by a red frame.
$ICNO_3$ = intracellular nitrate

Table S3 | Non-parametric correlation analysis of N-turnover versus time for incubations of diatom-bacteria aggregates prepared with $^{15}NO_3^-$-storing *S. marinoi* (Fig. 4).

	$ICNO_3$-derived N-turnover				$ECNO_3$-derived N-turnover			
Time interval	NO_3^-	NO_2^-	NH_4^+	N_2	NO_3^-	NO_2^-	NH_4^+	N_2
0-6 h	*R* = 0.527<br>*p* = 0.15	*R* = 0.158<br>*p* = 0.69	*R* = 0.632<br>*p* = 0.07	*R* = –0.105<br>*p* = 0.79	*R* = –0.316<br>*p* = 0.41	*R* = 0.422<br>*p* = 0.26	*R* = –0.105<br>*p* = 0.79	*R* = 0.106<br>*p* = 0.79
6-48 h	*R* = –0.949<br>*p* < 0.01	*R* = 0.896<br>*p* < 0.01	*R* = 0.896<br>*p* < 0.01	*R* = 0.949<br>*p* < 0.01	*R* = –0.791<br>*p* = 0.01	*R* = 0.949<br>*p* < 0.01	*R* = 0.211<br>*p* = 0.59	*R* = 0.949<br>*p* < 0.01

Positive and negative Spearman's rank coefficients *R* indicate net production and consumption of a given N-compound, respectively.
Significance levels $p < 0.05$ indicate that the net turnover of a given N-compound is significantly different from zero.
Significant correlations are marked by a red frame.
$ICNO_3$ = intracellular nitrate, $ECNO_3$ = extracellular nitrate
